# Antioxidant Activity of Sweet Whey Derived from Bovine, Ovine and Caprine Milk Obtained from Various Small-Scale Cheese Plants in Greece before and after In Vitro Simulated Gastrointestinal Digestion

**DOI:** 10.3390/antiox12091676

**Published:** 2023-08-27

**Authors:** Eleni Dalaka, Ioannis Politis, Georgios Theodorou

**Affiliations:** Laboratory of Animal Breeding and Husbandry, Department of Animal Science, Agricultural University of Athens, 11855 Athens, Greece; elenidalaka@aua.gr (E.D.); i.politis@aua.gr (I.P.)

**Keywords:** cheese whey, in vitro digestion, bioactive peptides, antioxidant biochemical assays, cellular assays

## Abstract

Whey-derived peptides have been associated with different biological properties, but most peptides are usually further hydrolyzed during the digestive process. In the present study, the antioxidant capacity of 48 samples of sweet whey (SW) derived from cheeses obtained from small-scale cheese plants made with bovine, ovine, caprine or a mixture of ovine/caprine milk was assessed using both cell-free and cell-based assays. SW digestates (SW-Ds) and a fraction (<3 kDa; SW-D-P3) thereof were obtained after in vitro digestion and subsequent ultrafiltration. Antioxidant properties using four different assays were evaluated before and after digestion. Our data showed higher values (*p* < 0.05) for ORAC, ABTS, FRAP and P-FRAP after in vitro digestion (SW-Ds and SW-D-P3) when compared with the corresponding values before digestion. In the non-digested SW, ORAC values were higher (*p* < 0.05) for the bovine SW compared with all the other samples. In contrast, the ABTS assay indicated a higher antioxidant activity for the ovine SW both before digestion and for SW-D-P3 compared with the bovine SW. The fraction SW-D-P3 of the ovine SW, using HT29 cells and H_2_O_2_ as an oxidizing agent, increased (*p* < 0.05) the cellular antioxidant activity. Furthermore, the same fraction of the ovine/caprine mixed SW increased, through the NF-κB pathway, the expression of SOD1 and CAT, genes implicated in the oxidative response in macrophage-like THP-1 cells. These findings indicate that SW, and particularly bovine and ovine SW, could be a candidate source for physical antioxidants in human and animal nutrition.

## 1. Introduction

Sweet whey (SW), a by-product of the cheese-making process, is considered a pollutant, and its estimated annual production is about 200 million tons [1]. Considering the huge volumes of SW produced and the increasing consumer demand for more sustainable food production, constant efforts are being made to evaluate biologically relevant properties of whey [2].

The demand for functional foods has been growing continuously due to their nutritional value, health-promoting properties and their potential to reduce the risk of several diseases [3,4]. Whey proteins are a source of bioactive peptides with a range of biological properties including antihypertensive, antimicrobial, antidiabetic as well as antioxidant activity. Specifically, whey proteins and their derivatives are characterized as potent antioxidants by modulating a range of redox biomarkers and reactive oxygen species [5,6]. The antioxidant activity of whey protein peptides has been attributed to the synergistic action of sulfhydryl groups, free radical scavenging by specific amino acids and chelation of iron [7,8]. It should be noted that although there is a plethora of studies reporting milk whey antioxidant activity, most of them use as a source WPC and WPI of bovine origin and not fresh SW, while no comparative data with SW from small ruminants’ milk are available.

During the last few years, much research has been dedicated to the processing and generation of bioactive peptides from food products [9]. Bioactive peptides are defined as components with biological activities over and above their nutritional value [10]. Furthermore, as it is known, whey proteins can release bioactive peptides by proteolysis in order to exert enhanced functions [11]. Polypeptides are degraded amongst other ways, by brush-border or cellular peptidases, whereas peptides with low molecular weight may remain intact and exert their activity at the tissue level [12].

Several recent studies have focused on cell-based assays as a more appropriate technique that serves as an intermediate between in vitro biochemical assays and in vivo clinical trials in animals or humans. Wolfe et al. developed a quantitative cellular antioxidant activity (CAA) assay based on human hepatocarcinoma HepG2 cells for quantifying the antioxidant activity of phytochemicals, food extracts and dietary supplements [13]. Liver cells, however, cannot be considered the ideal in vitro model for measuring the efficacy of dietary antioxidants. On the other hand, many studies mention a good correlation between the in vitro absorption in intestinal (Caco-2 and/or HT29) cellular models and in vivo intestinal absorption; hence, they are being widely used as effective tools for predicting the human intestinal absorption of food compounds and drugs [14,15,16]. For this reason, Wan et al. developed a Caco-2 CAA assay for the quantitative evaluation of antioxidants [17]. This was further validated by Kellett et al. who reported more robust results for CAA in a Caco-2 cell model compared to HepG2 cells and who verified that epithelial cell lines, known to be good models of the intestinal barrier, are more appropriate cell models to determine the antioxidant activity for phenolic antioxidants [18]. Many studies mention a good correlation between the in vitro absorption in intestinal (Caco-2 and/or HT29) cellular models and in vivo intestinal absorption; hence, they are being widely used as effective tools for predicting human intestinal absorption of food compounds and drugs [14,15,16].

The enzymatic hydrolysis of whey protein concentrate (WPC) and whey protein isolate (WPI) can produce antioxidant peptides, and hence, functional additives based on them could be produced. An advantageous approach for both dairy industries and livestock farmers should be the utilization of natural compounds, such as SW and its derivatives. Therefore, it is imperative to evaluate SW for its antioxidant capacity in vitro for the potential future increase of their use in animal nutrition [6,19].

The overproduction of ROS leads to oxidative stress and promotes inflammation by the NFE2L2 (NFE2 Like BZIP Transcription Factor 2) antioxidant pathway [20]. NFE2L2 is well recognized for its critical role in the response to oxidative stress and its binding to antioxidant responsive elements (AREs) and the subsequent regulation of their expression [21,22,23]. Enzymes such as catalase (CAT) and superoxide dismutase 1 (SOD1) are used as markers to reflect the antioxidant status of the cell [24]. More specifically, CAT and SOD1 represent the indirect antioxidant activity that reflects the removal of ROS. SOD1 decomposes superoxide anion to H_2_O_2_, which CAT then converts into water and oxygen [25], and the expression of both enzymes is regulated by AREs, which have been previously activated by NFE2L2 [26]. Another pathway that has been proposed as an alternative to NFE2L2 is that of the transcription nuclear factor kappa-light-chain-enhancer of activated B cells (NF-κB). The expression of antioxidant enzymes, among them CAT and SOD1, might also be regulated by the NF-κB pathway [27].

The aim of this study was to evaluate the antioxidant capacity of commercial SW from ovine, bovine, caprine and a mix of ovine/caprine milk origin using the harmonized static in vitro digestion protocol INFOGEST 2.0 [28]. The oxygen radical antioxidant capacity (ORAC), 2,2′-azinobis (3-ethylbenzothiazoline-6-sulfonic acid) radical scavenging assay (ABTS), ferric reducing antioxidant power (FRAP) and potassium ferricyanide reducing antioxidant power (P-FRAP) of SW before and after digestion were examined. Moreover, the effect of treatment with SW-D-P3 on CAA in HT29 cells and gene expression quantification of CAT, SOD and NFE2L2 in THP-1 cells, following induced oxidative stress by H_2_O_2_ and LPS, respectively, was assessed.

## 2. Materials and Methods

### 2.1. Chemicals and Reagents

The chemicals and enzymes used were all of high purity or an analytical reagent grade. Trolox (6-hydroxy-2,5,7,8-tetramethylchroman-2-carboxylic acid), 2,2′-azobis(2-methyl-propionamidine) dihydrochloride (AAPH), 2,4,6-tri (2-pyridyl)-*s*-triazine (TPTZ), fluorescein sodium salt (FL), ferric chloride (FeCl_3_), potassium ferricyanide (K_3_Fe(CN)_6_), trichloroacetic acid (TCA) and 2′,7′-Dichlorofluorescein diacetate (DCFH-DA), hydrogen peroxide (H_2_O_2_—35% in water), 2,2′-azino-bis(3-ethylbenzothiazoline-6-sulfonic acid (ABTS), sodium persulfate, acetic acid (glacial), butylated hydroxytoluene (BHT), pepsin from porcine gastric mucosa (≥2.500 units/mg protein), porcine pancreatin (4 × USP, United States Pharmacopeia) bile extract porcine, phorbol 12-myristate 13-acetate (PMA) and lipopolysaccharides from *Escherichia coli* O111:B4 (LPS) were all purchased from Sigma-Aldrich (Saint Louis, MO, USA). The Amicon Ultra-4 Centrifugal Filter Devices (3kDa) and Millex-GP 33mm PES 0.22 um were from Merck Millipore (Burlington, MA, USA). The 96-well transparent flat-bottom plates were purchased from Kisker Biotech (Steinfurt, Germany), while the 96-well cell culture transparent flat-bottom plates were purchased from SPL Life Sciences (Pocheon, South Korea). 3-(4,5-dimethylthiazol2-yl)-2,5-diphenyltetrazolium bromide (MTT) was obtained from Cayman Chemical (Michigan, MI, USA). DMEM, RPMI 1640, L-glutamine, sodium pyruvate, non-essential amino acids and penicillin-streptomycin were purchased from Biosera (Cholet, France). Fetal bovine serum (FBS) was purchased from Gibco ThermoFisher Scientific (Waltham, MA, USA). Phosphate-buffered saline (PBS) and the PrimeScript RT reagent kit (Perfect Real Time) were purchased from Takara Bio (Shiga, Japan). NucleoZOL was obtained from Macherey-Nagel (Düren, Germany). DNase I (RNase-Free) was purchased from New England Biolabs (Ipswich, MA, USA). The FastGene 2× IC Green qPCR Universal Mix was purchased from Nippon Genetics (Tokyo, Japan).

### 2.2. Collection and Preparation of Samples

Sweet whey samples were obtained from several small-scale cheese plants in Greece. Cheeses were produced from bovine, ovine, caprine and a mixture of ovine/caprine milk. An equal number (12 each) of SW from each milk animal origin (bovine, ovine, caprine and a mix of ovine/caprine) was used. The ratio of ovine/caprine ranged from 80/20 to 70/30 in all samples used in this study. Freeze-drying of liquid sweet whey was carried out to remove water and other solvents, and then protein content of all 48 samples was determined by the Kjeldahl method [29]. Afterwards, all samples were resuspended in deionized water at the same protein concentration of 3% (*v*/*v*) before use in subsequent analyses.

### 2.3. In Vitro Digestion Protocol

The digestion procedure of the samples was modified using the protocol reported by the amended and improved digestion method INFOGEST 2.0 [28,29] with slight modifications. Electrolyte stock solutions of digestive fluids, including the simulated salivary fluid (SSF), simulated gastric fluid (SGF) and simulated intestinal fluid (SIF), were prepared, and the pH of the electrolyte stock solutions were adjusted with HCl (5 M) and NaOH (6 M). Enzyme and bile salt solutions were freshly prepared prior to use.

#### 2.3.1. Simulated Oral Digestion

Briefly, 5 mL of each sample (30 mg protein/mL) was mixed with 4 mL of pre-warmed SSF followed by the addition of 25 μL of CaCl_2_ (0.3 M) and 0.975 mL of distilled water and NaOH (1 M) to reach a pH of 7. The oral solution was placed in a water bath at 37 °C and incubated with shaking for 2 min without the addition of α-salivary amylase.

#### 2.3.2. Simulated Gastric Digestion

A total of 10 mL of the above oral bolus was mixed with 8 mL of pre-warmed SGF, 1 mL of porcine pepsin solution (2000 U/mL), 5 μL of CaCl_2_ (0.3 M) and 0.995 mL of distilled water with HCl (5 M) to achieve a pH of 3. The final mixture was incubated for 2 h at 37 °C in a rotator at 300 rpm.

#### 2.3.3. Simulated Intestinal Digestion

A total of 20 mL of the gastric chyme was mixed with 8 mL of pre-warmed SIF, 5 mL of pancreatin (100 U/mL), 2.87 mL of bile extract (5 mM), 40 μL of CaCl_2_ (0.3 M) and 4.09 mL of distilled water with NaOH. The final pH of each mixture was adjusted to 7 to simulate the physiological intestinal digestion environment. Then, the final mixture was incubated for 2 h at 37 °C in a rotator at 300 rpm.

#### 2.3.4. Digestates’ Fractionation

SW digestates (SW-Ds) were immediately placed in a water bath for heat shock (85 °C, 10 min) to stop enzymatic activities and were then directly placed on ice. Undigested proteins were precipitated by centrifugation at 1200× *g* for 5 min. Then, aliquots of the digestates were filtered through a 0.22 μm polyvinylidene fluoride (PVDF) syringe filter. In order to remove high molecular weight peptides from SW-Ds and obtain the SW-D-P3 fraction, a membrane filter (Ultracel^®^ low binding regenerated cellulose) with an MWCO of 3 kDa was used (Amicon Ultra-4 Centrifugal Filter Devices). The SW-D samples were centrifuged at 4000× *g* for 90 min in a swinging-bucket rotor, following the manufacturer’s instructions. The SW-Ds and SW-D-P3 were then stored at −20 °C until needed for further analysis. Moreover, four replicates of blank digests were also prepared. Specifically, digests without SW (replaced with water) but with all required enzymes, electrolytes and bile salts were performed. The corresponding fractions after in vitro digestion were named BL-D for blank digest and BL-D-P3 for the fraction of digestate with peptides with a molecular weight below 3 kDa.

### 2.4. Biochemical Assays

#### 2.4.1. Oxygen Radical Antioxidant Capacity (ORAC)

The ORAC method used with FL was first described by Ou et al. [30], and the improved method of Zulueta and colleagues [31,32] was used. Firstly, the SW, SW-Ds and SW-D-P3 were dissolved and diluted 1:30 in PBS (75 mM, pH 7.4) so as to be transparent and to avoid interferences. A total of 20 μL of each sample was added to a well in a 96-well microplate with 120 μL FL (117 nM in 75 mM PBS, pH 7.4). After incubation with shaking at 37 °C for 15 min, 60 μL AAPH (40 mM) was also added to each well. Fluorescence was read immediately every 2 min for a total of 80 min at 485 nm/535 nm (excitation/emission). The automated ORAC assay was carried out on a VICTOR 2030 multilabel counter (Perkin Elmer, Waltham, MA, USA), and a standard curve of Trolox was constructed with a final concentration range of 3.125–50 μM in 75 mM of PBS and a pH of 7.4. The antioxidant capacity was expressed as μmol Trolox Equivalents (TEs)/g protein based on the area under curve (AUC) for the fluorescence decay vs. time. SW before and after in vitro digestion were measured in triplicate, and the experiment was performed three independent times.

#### 2.4.2. 2,2′-Azinobis (3-Ethylbenzothiazoline-6-Sulfonic Acid) Radical Scavenging Assay (ABTS)

The ABTS radical scavenging activity of the SW samples, before and after digestion, was measured according to the procedure of Ozgen et al. [33]. Firstly, 7 mM of an ABTS solution was mixed with 2.45 mM of a sodium persulfate solution in equal volumes, followed by incubation for 12–16 h at room temperature in the dark to generate ABTS radicals. After that, the above ABTS^•+^ solution was diluted with a sodium acetate buffer (20 mM, pH 4.5) until the absorbance provided nearly 0.7 at 734 nm. Then, 20 μL (3 mg protein/mL) of the sample and 230 μL of the ABTS^•+^ solution was mixed and incubated for 60 min at room temperature [4]. A standard curve of the antioxidant (Trolox) was constructed for a concentration range of 3.75–100 μM. The absorbance was measured at 734 nm using a 96-well Inifinite M200 Pro plate reader (Männedorf, Switzerland). The ABTS radical scavenging activity (%) was calculated as an ABTS radical scavenging activity (%) = [(Ac − As)/Ac] × 100 where Ac represents the absorbance of the control and As represents the absorbance of the samples after the reaction [34]. The SW samples before and after in vitro digestion were measured in triplicate, and the experiment was performed three independent times. The results are expressed as μmol TEs/g protein.

#### 2.4.3. Ferric Reducing Antioxidant Power (FRAP)

The reducing activity of SW, SW-Ds and SW-D-P3 was determined according to the method of Benzie et al. [35] with appropriate modifications for 96-well microplates [36,37]. Firstly, the SW was suitably diluted at 1:3 and SW-Ds and SW-D-P3 at 1:2, respectively. A ferric-tripyridyltriazine (Fe^III^-TPTZ) complex solution was prepared with 0.3 M sodium acetate with glacial acetic acid (pH 3,6), 20 mM FeCl_3_ and 10 mM TPTZ (in 40 mM HCl) at a ratio of 10:1:1 (*v*/*v*/*v*) and was heated to 37 °C for 1 h. All stock solutions were prepared daily and kept in the dark. Consequently, 280 μL of the Fe^III^-TPTZ solution was transferred to each well with 20 μL of the sample in a 96-well microplate, and the absorbance was read at 590 nm using the Epoch 2 microplate spectrophotometer (Biotek, Winooski, VT, USA). Trolox as standard (concentration range 0.18–5.88 μM) and methanol as blank were also added to each plate. Results are expressed as μmol TEs/g protein. Samples were measured in triplicate, and the experiment was performed three independent times.

#### 2.4.4. Potassium Ferricyanide Reducing Power (P-FRAP)

The reducing power assay was performed according to the method described by Oyaizu [38] and Liang et al. [39] with some modifications. Briefly, the SW, SW-Ds and SW-D-P3 were appropriately diluted with water (1:6 for all samples) so that the final absorbance of 700 nm fell within the range of the calibration curve. Then, 50 μL of the sample solution were mixed with 50 μL of 0.2 M of PBS (pH 6.6) and 50 μL of a fresh (prepared daily) K_3_Fe(CN)_6_ (1% *w*/*v*) solution. The microplate was incubated for 20 min at 50 °C under agitation. Then, 50 μL of TCA (10% *w*/*v*) with 10 μL of FeCl_3_ (0.1% *w*/*v*) were added, and the incubation was continued for an additional 10 min at 50 °C under agitation. The absorbance at 700 nm was measured, and a curve with a BHT was constructed (0–100 μM). Results are expressed as μmol BHT equivalents/g protein [40]. The reducing power assay measures the ability of a compound to reduce ferric ion (Fe^3+^) to ferrous ion (Fe^2+^) through electron or hydrogen donation [41]. Samples were measured in triplicate, and the experiment was performed three independent times.

### 2.5. Cellular Assays

#### 2.5.1. Cell Culture and Cell Viability of HT29

Cells of the human colon adenocarcinoma cell line HT29 were cultured in DMEM supplemented with 100 U/mL of penicillin, 100 μg/mL of streptomycin, 10 U/mL of L-glutamine, 100 μM of non-essential amino acids, 1 mM of sodium pyruvate and 10% (*v*/*v*) FBS. Cells were incubated at 37 °C in humidified air containing 5% CO_2_. Firstly, the effects of H_2_O_2_ on cell survival were measured using the MTT assay. The HT29 cells were seeded in 96-well plates at a concentration of 0.5 × 10^5^ cells per well. After 24 h of culturing, the cells were treated with different concentrations of H_2_O_2_ (0–2 mM) at 37 °C. The following day, the cells were washed twice with PBS, incubated for an additional 2–3 h at 37 °C with MTT (0.5 mg/mL). Then, the supernatants were removed and 100 μL of DMSO was added. The absorbance was quantified at a 570 nm wavelength (Tecan Infinite^®^ M200 PRO). Results are expressed as a percentage of the untreated control cells (without H_2_O_2_). Each point represents the mean of two experiments with each individual treatment being run in quadruplicate. Furthermore, different concentrations (0.75–6 mg protein/mL) of the SW-D-P3 fraction in the presence of 0,5 mM of H_2_O_2_ were evaluated for their cytotoxic effects in HT29 by the MTT assay.

#### 2.5.2. Cellular Antioxidant Activity (CAA) Assay

CAA indicates the overall oxidative status by monitoring the decomposition of DCFH-DA in the cells and its oxidation by reactive oxygen species (ROS) into the fluorescent DCF [42,43]. Intracellular reactive oxygen species (ROS) levels were determined in the HT29 cells as described by Piccolomini et al. [43] and adapted by Feng et al. [44] with some modifications. Cells were seeded at 0.5 × 10^5^ cells/well in 96-well plates for 24 h. Cells were washed with PBS and treated with 50 μL of BL-D-P3 or SW-D-P3 (6 mg protein/mL in DMEM) together with 50 μL of H_2_O_2_ (0.5 mM in DMEM) for 24 h. Afterwards, the cells were washed twice with PBS and treated with 100 μL of DCFH-DA (10 μM in PBS containing 0.2% methanol) for 30 min. Fluorescence at 485 nm/535 nm was recorded at 37 °C every 2 min for a total of 80 min using the VICTOR 2030 multilabel counter (Perkin Elmer, Waltham, MA, USA). CAA was measured in triplicate, and the experiment was performed three independent times. The results are expressed as a % of ROS generation to the untreated cells (without H_2_O_2_).

#### 2.5.3. Cell Culture and Differentiation of THP-1

Cells of the human acute monocytic leukemia cell line THP-1 were maintained in RPMI 1640 supplemented with 10% (*v*/*v*) FBS, 10 U/mL of L-glutamine, 1 mM of sodium pyruvate, 100 U/mL of penicillin, 100 μg/mL of streptomycin and 100 μM of non-essential amino acids in a humidified incubator at 37 °C and 5% CO_2_. To induce differentiation into macrophage-like ones, monocytes were placed into 12-well plates at a cell density of 0.8 × 10^6^ cells/mL and incubated with 100 ng/mL of PMA for 48 h [45,46,47,48]. Then, the PMA-contained medium was removed, and cells were washed with PBS and subsequently incubated for 24 h in the supplemented PMA-free RPMI-1640. After the resting phase, macrophages were incubated for 24 h in the presence of 100 ng/mL of lipopolysaccharide (LPS) and SW-D-P3 (3 mg protein/mL) or BL-D-P3 for 24 h. Each sample was tested in triplicate.

#### 2.5.4. Quantification of mRNA Transcripts Using Real Time-PCR (qPCR)

Total RNA extraction of the attached THP-1 cells was performed using the Nucleozol reagent according to the manufacturer’s instructions. RNA samples were treated with DNase for the removal of the remaining DNA, and pure RNA was recovered by ethanol precipitation. The quantity and purity of RNA was calculated using a spectrophotometer (Q5000, Quawell Technology Inc., San Jose, CA, USA). Reverse transcription was performed from 500 ng of the total RNA with the PrimeScript RT reagent kit following the protocol of the manufacturer. A Real-Time thermal cycler (SaCycle–96, Sacace Biotechnologies, Como, Italy) was used for the qPCR using the FastGene 2× IC Green qPCR Universal Mix. Primers for target genes (*SOD1*, *CAT*, *NFE2L2*, *NFKB1* and *RELA*) and housekeeping genes (*RPS18*, *HPRT1*, *RPL37A* and *B2M*) were designed across intron/exon boundaries with an annealing temperature of 60 °C. Each cDNA sample was tested in duplicate. The relative gene expression was calculated using the 2^−ΔΔCt^ method. Primer details are listed in Table 1.

### 2.6. Statistical Analysis

The experimental results are reported as means ± the standard error of means (SEMs) of at least two biological replicates. All data were tested for normality using the Kolmogorov–Smirnov test and transformed in logarithmic or normalized form [49] where necessary until the data were normally distributed. Subsequently all data generated were compared using one-way ANOVA followed by Duncan’s post hoc test. Differences between means were considered significant at *p* < 0.05. The statistical analysis was performed using the SPSS for Windows statistical package program, version 22.0.0. Graphs were generated using the GraphPad Prism 8 program.

## 3. Results and Discussion

To evaluate whether gastrointestinal digestion affects the antioxidant activity of SW, the standardized INFOGEST static in vitro digestion model was applied. The antioxidant properties of SW, SW-Ds and SW-D-P3 were evaluated using a variety of methodological approaches. Due to the differences in the principle and mechanism of action of the different methods of antioxidant capacity evaluation, the use of only one antioxidant analysis can barely clarify the actual antioxidant status of samples [50]. Consequently, four different biochemical antioxidant methods are employed towards this aim, which can roughly be classified into two types, namely, assays based on hydrogen atom transfer (HAT) reactions and those based on single electron transfer (SET) [51]. HAT assays, such as ORAC, measure the ability of an antioxidant to inactivate a free radical (ROO·) by releasing a hydrogen atom in kinetic time. In contrast, assays that are dominated by SET-based reaction mechanisms, such as the end-point ABTS, measure the release of an electron to the (ROO·), converting it into an anion (ROO−) [52,53]. The latter causes a color change in the solution, indicating the concentration of the antioxidant. Thus, it was crucial to use at least one assay of each type in order to more completely evaluate the total antioxidant activity of a complex substrate such as SW. In addition, the cellular antioxidant activity of the SW-D-P3 fractions was evaluated using CAA in the HT29 intestinal cell line and expression of oxidative stress-related genes in the THP-1 monocytic cell line.

### 3.1. Assessment of Antioxidant Activity of SW before and after In Vitro Digestion Using ORAC, ABTS, FRAP and P-FRAP Biochemical Assays

The data shown in Table 2 indicate that the antioxidant activity of the samples after in vitro digestion was greatly increased, regardless of the biochemical method used. In all four assays, the antioxidant activity of SW was significantly increased SW-D>SW-D-P3>SW (*p* < 0.05). The digestive process had a positive effect on the antioxidant capacity of SW, represented by significant increases of around 2–4 fold on the ABTS, FRAP and P-FRAP assays after in vitro digestion, while an even greater augmentation was observed by the ORAC-FL assay (Table 2). In line with our results, there are other studies that report a significantly increased antioxidant activity of WPI after digestion, regardless of the assay used [37,54]. In addition, Garcia-Casas et al. [55] demonstrated that the bioaccessible fraction (corresponding to the SW-D samples of our study) of a SW-based beverage digestate exhibited an augmented antioxidant activity based on ABTS and FRAP. Since antioxidant compounds can use different mechanisms of action and each of the methods used evaluates their effect in a unique way, their evaluation of said compounds is complementary to each other. Namely, ABTS, FRAP and P-FRAP belong to the single-electron transfer (SET) assays while ORAC-FL belongs to the hydrogen atom transfer (HAT) assays. Interestingly, from our data in Table 2, the ORAC values showed a much-enhanced antioxidant activity for the SW samples after digestion, with an 8-fold increase, from 20.5 to 167.2 μmol TEs/g protein. Although Clausen et al. observed that the ORAC assay was more sensitive than ABTS in evaluating the scavenging of the peroxyl radicals of bovine whey proteins, it should be noted that this observation was made for intact whey proteins rather than fermented ones [56].

In Table 2, the values of P-FRAP show a significant increase (*p* < 0.05) of antioxidant activity after simulated gastrointestinal digestion (7.51 for SW to 26.9 μmol BHT eqv/g protein for SW-Ds). The results of the increased P-FRAP values after gastrointestinal digestion are in accordance with a previous study by Shaukat et al. on buffalo milk-processed cheddar cheese [57].

The overall observed increase in antioxidant capacity after digestion could be attributed to the release of peptides and free amino acids by the simulated gastrointestinal digestion [11]. Moreover, since SW results from a fermentation procedure, this could further enhance the release of antioxidant bioactive peptides [58].

Peptides released during an enzymatic hydrolysis process have considerable variability in size and structural characteristics [59], with peptides of relatively low molecular weight tending to display a relatively high antioxidant capacity [60,61]. Besides gastrointestinal digestion, such fragments can be also produced technologically using a broad range of exogenously supplied enzymes such as alcalase, chymotrypsin and flavourzyme [11,62]. From the data in Table 2, it is evident that the SW-D-P3 fraction accounts for the majority (65 to 85%) of the antioxidant capacity of the SW-Ds, regardless of the assay used. This indicates that the peptides with antioxidant activity are predominantly of lower molecular weight. Consistent with our results, Athira et al. reported that the antioxidant activity of WPC alcalase hydrolysed permeate (3 kDa) was augmented compared to WPC [63]. Similarly, another study indicated that the peptides between 0.1 to 2.8 kDa of WPI alcalase hydrolysate displayed the strongest radical scavenging activity [64]. Furthermore, O’Keeffe and Fitzgerald showed a greater antioxidant activity of WPC hydrolysate fractions with low molecular mass peptides (<5 kDa and <1 kDa) than WPC as measured by ORAC [65]. Also, a recent study of Ballatore et al. reported the highest antioxidant activity for the <3 kDa fraction obtained from trypsin-hydrolyzed WPC [19].

#### Effect of Milk Animal Origin

**Figure 1:**
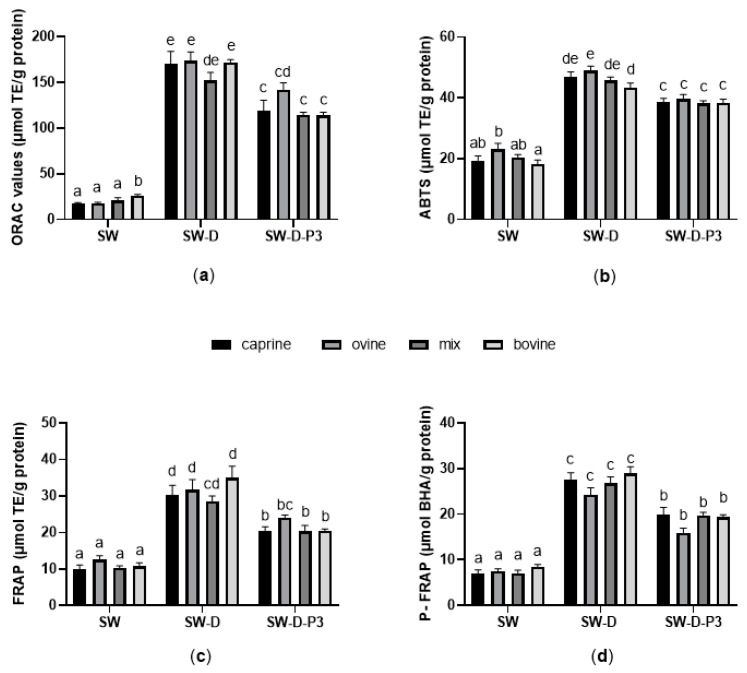
Antioxidant activity of sweet whey before (SW) and after in vitro digestion (digested SW (SW-Ds) and digested fraction below 3 kDa (SW-D-P3)) assessed with (**a**) oxygen radical absorbance capacity assay, expressed as μmol of TEs per gram of protein; (**b**) 2,2′-Azinobis (3- ethylbenzothiazoline-6-sulfonic acid), expressed as μmol of TE per gram of protein; (**c**) ferric reducing antioxidant power assay, expressed as μmol of TE per gram of protein; and (**d**) potassium ferricyanide reducing power, expressed as μmol of BHA per gram of protein. Results represent the mean of 3 experimental repetitions ± SEM (*n* = 12). Columns with different letters within the same panel are significantly different (*p* < 0.05).

The antioxidant activities with the ORAC and ABTS assays for bovine, ovine, caprine and a mix of ovine and caprine of SW, SW-Ds and SW-D-P3 are shown in Figure 1, panels a and b, respectively. The ORAC values of antioxidant activity were highest for bovine SW (26.0 μmol TEs/g protein) compared with the other three groups (*p* < 0.05), whereas after in vitro digestion (SW-Ds and SW-D-P3), no significant differences were observed (*p* > 0.05). In contrast, the ABTS values of ovine SW were higher, compared to their bovine counterparts, both before (23.2 μmol TEs/g protein) and after in vitro digestion (49.0 μmol TEs/g protein for the SW-Ds). There were no statistically significant differences (*p* > 0.05) in the FRAP and P-FRAP values between the SW samples derived from cheeses made from the four different milk sources (Figure 1c,d, respectively).

### 3.2. Assessment of Cellular Antioxidant Activity of SW-D-P3

Food digestates are known to be cytotoxic to HT29 cells [66]. Thus, the HT29 cells were first exposed for 24 h to a range of concentrations of SW-D-P3 (0.75–6 mg protein/mL) to assess their cytotoxic effect. The MTT assay indicated that none of the concentration was cytotoxic. It was inferred that the concentration of the samples used in our study would not influence the activity of HT29 cells and thus would not affect the result of CAA. Therefore, the CAA assay was performed on the SW-D-P3 of a maximum concentration of 6 mg protein/mL. This concentration is in the range used in a previous study. In more detail, Kleekayai et al. evaluated the ROS generation in stressed HepG2 cells treated with hydrolysates of WPC at concentrations ranging from 0 to 10 mg/mL [67]. The highest concentration (10 mg/mL) exhibited the most potent cellular ROS generation reducing activity in the intracellular ROS generation in AAPH-stressed cells. Other studies reported a dose–response augmentation of CAA with the supplementation of whey proteins in different cell lines and are presented thoroughly in a review by Corrochano et al. [6].

**Figure 2:**
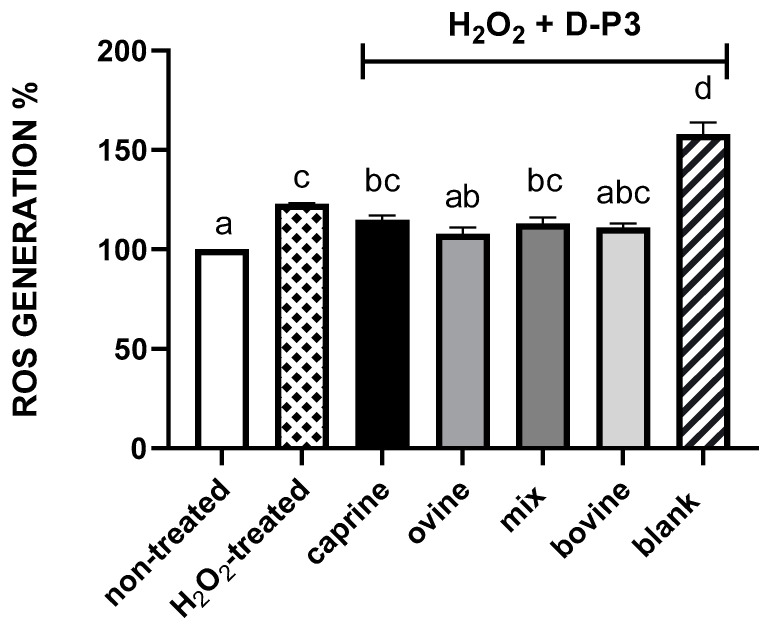
Reactive oxygen species (ROS)% generation in HT29 cells (seeded at 5 × 10^4^ cells/well) after 24 h exposure to sweet whey after gastrointestinal digestion with peptides below 3 kDa (SW-D-P3) at a concentration of 6 mg protein/mL. Non-treated cells = cells without H_2_O_2_, H_2_O_2_-treated = cells treated with 0.5 mM of H_2_O_2_, caprine, ovine, bovine and a mix of caprine/ovine = SW-D-P3 and blank = BL-D-P3, all treated with 0.5 mM H_2_O_2_. Cell treatment was performed in triplicate on three different days, and the values reported are mean ± SEM. Columns with different letters are significantly different (*p* < 0.05).

The cell cytotoxicity of the oxidative stress inducer, H_2_O_2_, was also pre-evaluated at concentrations ranging from 0–2 mM in order to investigate their potential toxic effects on HT29 cells (Supplementary Data, Figure S1). A toxic effect yielding <80% cell viability was found at levels >1 mM. Due to the similar effects observed for H_2_O_2_ at concentrations below 1 mM, a concentration of 0.5 mM of H_2_O_2_ was selected to represent the oxidative stress inducer. Previous studies have reported the same range of H_2_O_2_ used as an oxidative stress inducer (0.25–0.7 mM) to evaluate the protective effect of food compounds [43,44,68,69]. Several studies have linked low-molecular size peptides with an improved antioxidant activity [64,70,71]. From our results in biochemical assays, it was concluded that the SW-D-P3 fraction was the one responsible for the majority of the antioxidant activity of digested SW. Therefore, in the present work, the HT29 epithelial cell line was used as a cellular model for the estimation of ROS% inhibition by SW-D-P3. The results presented in Figure 2 show that cell treatment with 6 mg protein/mL of SW-D-P3 significantly reduced radical formation in H_2_O_2_-treated cells, regardless of the SW milk origin, compared to the BL-D-P3 (*p* < 0.05). Furthermore, cell treatment with ovine SW-D-P3 significantly reduced the radical formation cells compared to the H_2_O_2_-treated group (*p* < 0.05), with a decrease of 14.97%, while the levels of radical formation for the cells treated with ovine and bovine SW-D-P3 did not differ significantly with those of the cells not treated with H_2_O_2_ (*p* > 0.05).

The results from previous studies regarding CAA determination in food and feed components after in vitro digestion using cell-based assays in various cell lines are scarce and are predominantly focused on non-dairy products. More specifically, CAA was found to be higher in digested whole grains compared to the free fraction in the HepG2 cell line [72]. Moreover, chickpea protein hydrolysate inhibited DCFH oxidation in a dose–response manner, and an increased CAA unit was noticed at higher hydrolysate protein concentrations [73]. A previous study by Zhang et al. [74] reported a decrease in ROS% generation in Caco-2 cells treated with soy protein hydrolysate compared to the H_2_O_2_ group. In another study [75], the digested and fractionated eggshell membrane (ESM) hydrolysate were evaluated in Caco-2 cells with AAPH used as a radical generator. In detail, the CAA of the digested ESM hydrolysate fraction with molecular weight of <5 kDa was higher compared with fractions of 5–10 kDa and >10 kDa. To the best of our knowledge, there are only two studies reporting CAA in eukaryotic cells in dairy-associated compounds after in vitro digestion. In the first one by Corrochano et al. [76], b-LG and a-LA, after in vitro digestion, effectively inhibited ROS and stimulated antioxidant enzymes in HT29 cells, while the same whey proteins were unable to reduce induced ROS formation in Caco-2 cells. Moreover, it should be noted that contrary to our results, they did not observe such an effect in WPI digestates. On the other hand, the second of these studies is in agreement with our results, as a better cellular antioxidant activity of digested WPI using the Caco-2 cell line was reported in response to H_2_O_2_ [77]. Interestingly, using a non-eukaryotic cell model, Ibrahim et al. reported a protective effect of camel milk protein hydrolysates (casein and whey hydrolysates separately) on yeast cells against H_2_O_2_-induced oxidative stress [78].

### 3.3. Effect of SW-D-P3 on Expression of Antioxidant Genes

**Figure 3:**
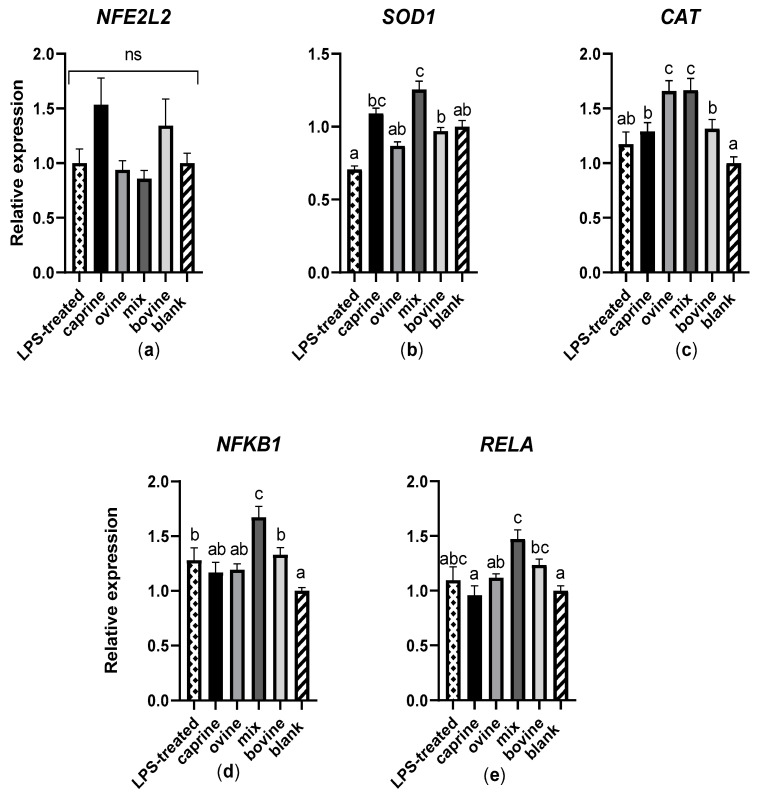
Effect of SW-D-P3 on LPS-induced mRNA expression in THP-1 cells. THP-1 cells were pretreated with PMA for 48 h (100 ng/mL), a 24 h rest and then were treated with LPS (100 ng/mL) with or without the presence of 3 mg protein/mL of SW-D-P3 (caprine, ovine, mix and bovine) or BL-D-P3 (blank) for 24 h. The expression levels of (**a**) *NFE2L2*, (**b**) *SOD1*, (**c**) *CAT*, (**d**) *NFKB1* and (**e**) *RELA* were measured using real-time PCR and were normalized to four housekeeping genes (*B2M*, *RPL37A*, *RPS18* and *HPRT1*). Data are represented as mean ± SEM of three independent determinations (*n* = 3). Columns with different letters within the same panel are significantly different (*p* < 0.05); ns = not significant (*p* > 0.05).

In the present study, the relative gene expression of NFE2L2, SOD1 and CAT, essential components of antioxidant signaling pathways, was measured in response to treatment with SW-D-P3 in LPS-challenged THP-1-derived macrophages. Additionally, the expression of *NFKB1* and *RELA* encoding the two main subunits of the NF-κB transcription factor was also quantified. The *CAT* expression was found to be higher in SW-D-P3-treated cells when compared to BL-D-P3, regardless of milk animal origin. This increase, however, was attenuated in the mixed and ovine samples when compared to the bovine and caprine ones (*p* < 0.05; Figure 3c). The *SOD1* expression in turn was higher (*p* < 0.05; Figure 3b) only in the mixed samples when compared with BL-D-P3 and with the ovine and bovine samples but not with the caprine ones. On the other hand, no statistically significant differences were observed between the samples or with the BL-D-P3 regarding the *NFE2L2* expression (*p* > 0.05; Figure 3a). Finally, the *NFKB1* and *RELA* expressions (*p* < 0.05; Figure 3d,e, respectively) were found to be higher in the bovine and mixed SW-D-P3 compared to BL-D-P3.

In line with our results, Xu et al. also reported that treatment with WPC increases the enzymatic activity of SOD1 and CAT, while, at the same time, no significant differences were found in the *NFE2L2* expression in the myoblast cell line C_2_C_12_ following H_2_O_2_ oxidative stress [79]. Kerasioti et al. reported similar findings for the effect of WPC on SOD1 and CAT enzymatic activities and protein levels and NFE2L2 protein levels in the same cell line without the use of an oxidative agent, while using the same experimental parameter treatment with WPC resulted also in the increase of NFE2L2 protein levels in EA.hy926 endothelial cells [80]. In another study, HepG2 cells were treated with various concentrations of a glucose–WPC conjugate for 24 h followed by t-BHP oxidative stress to evaluate the role of NFE2L2 in the maintenance of the cellular redox status, reporting a dose-dependent increase of *NFE2L2* mRNA levels [81]. Furthermore, both the glutathione and catalase antioxidant systems are activated by WPC hydrolysate supplementation in human umbilical vein endothelial cells (HUVECs), resulting in an increase in cellular glutathione and CAT activity, albeit without the use of an oxidative stress factor [65]. Moreover, Corrochano et al. reported lower mRNA levels of *CAT* and *SOD1* in Caco-2 cells after a 4 h exposure to gastrointestinal digested samples of WPI when compared to the digestion control (equivalent to the BL-D-P3 of the present study), though, once more, no oxidative stress factor was used [76].

Based on the data produced by the present study, the induction of the expression of SOD1 and CAT by SW-D-P3 seems to be independent of the NFE2L2 pathway and is rather directed by NF-κB. The NF-κB pathway has been implicated in the regulation of many antioxidant and pro-oxidant targets [27]. More specifically, NF-κB is identified as a positive regulator of *SOD1* [82]. On the other hand, the role of NF-κB in regulation of the *CAT* expression is rather unclear as there are contradicting results supporting its role both as a negative [83] and as a positive [84] regulator of *CAT*. Our findings regarding the effect of SW-D-P3 in LPS-activated THP-1 support the latter and furthermore confirm the role of NF-κB in the induction of the *SOD1* expression, although further experimentation is needed to clarify the precise mechanism of their regulation. Corrochano et al. quantified mRNA transcripts of the antioxidant genes *SOD1* and *CAT* in intestinal cells that had been treated with 2.5 mg/mL of gastrointestinal bovine whey proteins (corresponding to the SW-Ds in our study) [76]. A recent study by Ishikawa evaluated some genes (e.g., *IL6* and *IL10*) implicated in inflammation and immunosuppression in THP-1 cells stressed with LPS and treated with 5 mg/mL of a whey protein hydrolysate [85].

Human or animal intervention trials with diets including whey products are the best way to assess their potential antioxidant benefit. However, only a small number of studies have evaluated the antioxidant effect of whey proteins/peptides using in vivo models. Ebaid et al. observed that dietary supplementation with whey proteins enhances the normal inflammatory responses during wound healing in diabetic mice by restoring the levels of oxidative stress [86]. Furthermore, Athira et al. reported the ameliorative potential of whey protein hydrolysates against paracetamol-induced oxidative stress in mice, compared with mice without whey protein administration. A significant increase in liver CAT and SOD levels and a reduction of the concentrations of oxidative biomarkers, such as alkaline phosphatase and creatinine, was observed [87].

In general, to consider the physiological benefits of sweet whey, it is important to know that whey proteins do not reach the intestine in their intact form. Sousa et al. compared the total protein digestibility of WPI between in vitro and in vivo situations which resulted in a good correlation, with a tendency toward an overestimation for the in vitro approach [88]. The antioxidant capacity in commercial whey products, which are commonly used as food ingredients especially in the sports nutrition sector, is well known [6,89]. To the best of our knowledge, this is the first report in which SW from small-scale cheese plants is used as a raw material for evaluating antioxidant capacity before and after in vitro digestion. The protection produced by SW against in vitro-induced oxidative stress (biochemical and cellular assays) with different methodologies reveals that SW could be used in animal/human nutrition. As with all in vitro and cell culture experiments, there remains a possibility that these results may not translate to in vivo situations. Also, to address the limitations concerning the suitable concentration related to the clinical efficacy of SW, its physiological efficacy is necessary to be investigated in vivo, either in animal nutrition and/or human clinical trials.

## 4. Conclusions

In this research, SW of different milk origins was digested using a standardized static in vitro digestion method for mimicking monogastric gastrointestinal digestion. The samples were evaluated by multiple assays to test antioxidant activity, since a single assay is not sufficient to test all relevant factors affecting antioxidant capacity. All antioxidant properties of the SW-Ds were significantly higher than those of the intact SW. Of special interest was the evaluation of the antioxidant activity of SW-D-P3 directly in mammalian cell lines. Intestinal cell lines are the models proposed to better assess the in vitro antioxidant potential of a dietary compound. However, there is only a relatively small number of studies that employ cell culture models to assess the antioxidant potential of food and feed components. In conclusion, the present study showed that SW-D-P3 exerts an antioxidant effect both in epithelial and derived activated macrophage cell lines. Furthermore, a slightly better antioxidant capacity seems to be associated with bovine and ovine when compared to caprine SW. A validation of the observed differences could be achieved by in vivo animal studies or human clinical trials. Further investigation is also deemed necessary in order to determine the peptides’ sequences from SW with potential antioxidant activity in vivo.

## Figures and Tables

**Table 1:** Oligonucleotide primer sequences, amplicon size and reaction efficiency in qPCR.

Gene (Accesion Number)	Primer Direction	Sequence (5′-3′)	Amplicon Size	Reaction Efficiency
*SOD1* (NM_000454)	Forward	CGAGCAGAAGGAAAGTAATGG	194	95
	Reverse	CCAAGTCTCCAACATGCC		
*CAT* (NM_001752)	Forward	TGCCTATCCTGACACTCACC	137	92
	Reverse	GAGCACCACCCTGATTGTC		
*NFE2L2* (NM_001145412)	Forward	GATCTGCCAACTACTCCCA	121	90
	Reverse	GCCGAAGAAACCTCATTGTC		
*NFKB1* (NM_001165412)	Forward	GCACAAGGAGACATGAAACAG	189	97
	Reverse	CCCAGAGACCTCATAGTTGTC		
*RELA* (NM_001145138)	Forward	GGACTACGACCTGAATGCTG	228	105
	Reverse	ACCTCAATGTCCTCTTTCTGC		
*RPS18* (NM_022551)	Forward	CTGAGGATGAGGTGGAACG	240	98
	Reverse	CAGTGGTCTTGGTGTGCT		
*HPRT1* (NM_000194)	Forward	CTTTGCTTTCCTTGGTCAGG	111	99
	Reverse	CAAATCCAACAAAGTCTGGCT		
*RPL37A* (NM_000998)	Forward	AGTACACTTGCTCTTTCTGTGG	119	106
	Reverse	GGAAGTGGTATTGTACGTCCAG		
*B2M* (NM_004048)	Forward	GCTATCCAGCGTACTCCA	285	103
	Reverse	CTTAACTATCTTGGGCTGTGAC		

**Table 2:** Antioxidant activity by ABTS (2,2′-azinobis (3-ethylbenzothiazoline-6-sulfonic acid)), ORAC-FL (Oxygen Radical Absorbance Capacity-Fluorescence), FRAP (ferric reducing antioxidant power) and P-FRAP (potassium ferricyanide reducing power) of sweet whey before (SW) and after in vitro digestion (SW-Ds and SW-D-P3) regardless of milk origin.

Method (Units)	SW	SW-Ds	SW-D-P3
ABTS (μmol TEs/g protein)	20.3 ± 0.8 ^a^	46.2 ± 0.8 ^c^	38.7 ± 0.6 ^b^
ORAC-FL (μmol TEs/g protein)	20.5 ± 1.0 ^a^	167.2 ± 4.8 ^c^	122.3 ± 3.9 ^b^
FRAP (μmol TEs/g protein)	10.9 ± 0.5 ^a^	31.4 ± 1.3 ^c^	21.3 ± 0.6 ^b^
P-FRAP (μmol BHT eqv/g protein)	7.5 ± 0.3 ^a^	26.9 ± 0.8 ^c^	18.7 ± 0.6 ^b^

Values are means ± SEM (*n* = 48). Mean values in each row with different letters are significantly different (one-way ANOVA and Duncan test, *p* < 0.05).

## Data Availability

The data presented in this study are available on request from the corresponding author.

## Supplementary Materials

The following supporting information can be downloaded at: https://www.mdpi.com/article/10.3390/antiox12091676/s1, Figure S1: Effect of different concentrations of oxidative stress inducer, hydrogen peroxide (H_2_O_2_), on HT29 cell viability.
