# Supplementary material for: Antioxidant Activity of Sweet Whey Derived from Bovine, Ovine and Caprine Milk Obtained from Various Small-Scale Cheese Plants in Greece before and after In Vitro Simulated Gastrointestinal Digestion
Source: Antioxidants (Basel). 2023 Aug 27;12(9):1676. doi: 10.3390/antiox12091676 (PMC10525972; doi:10.3390/antiox12091676)
Supplement: Supplementary file 1 [file antioxidants-12-01676-s001.zip › antioxidants-2527653-supplementary.pdf]

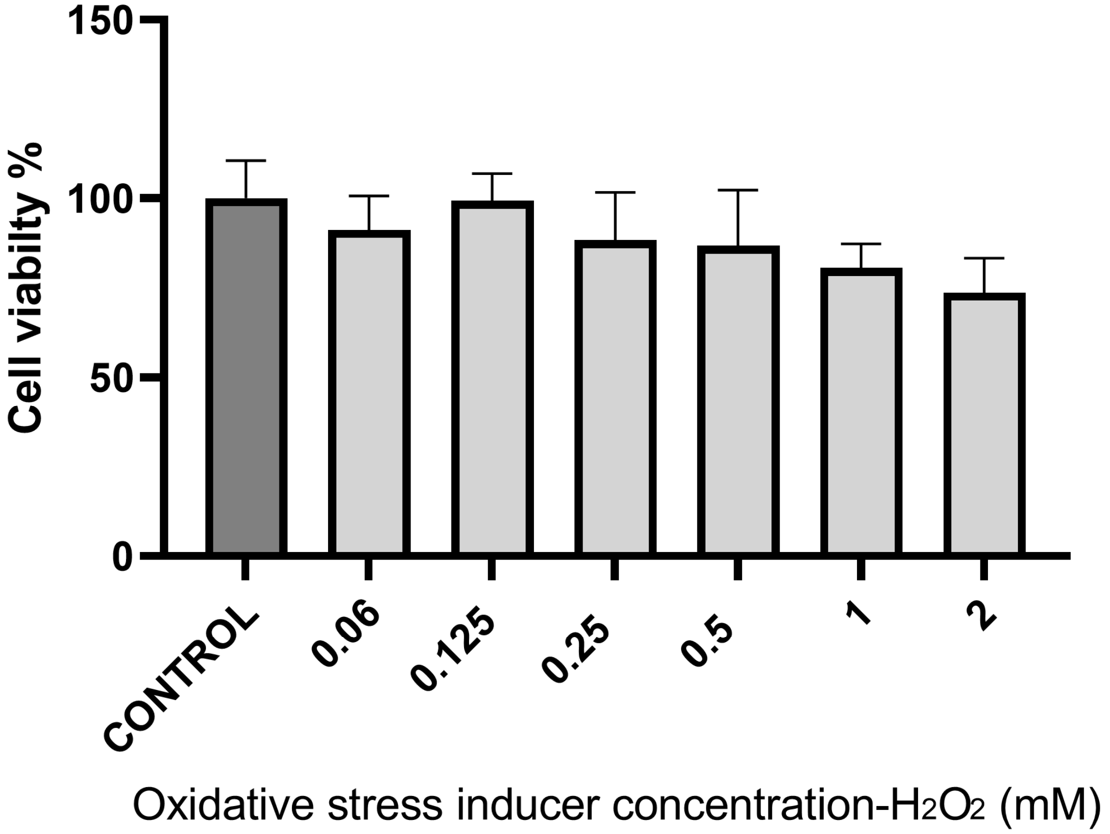

Figure S1. Effect of different concentrations of oxidative stress inducer, hydrogen peroxide (H_2O_2), on HT29 cell viability. The results are expressed as the percentage of viable cells remaining following treatment with oxidative stress inducer compared to untreated control cells (without H_2O_2). Values represent mean ± SD (n = 8).
